# Hospital Meetings, &c.

**Published:** 1899-05-20

**Authors:** 


### The Institutional Workshop.

### HOSPITAL MEETINGS, &C.

### SEAMEN'S HOSPITAL SOCIETY.

Establishment of the London School of Tropical Medicine.

Speech by Mr. Chamberlain.

The festival dinner of the Seamen's Hospital Society, for the purpose of establishing the London School of Tropical Medicine and the enlargement of the Branch Hospital in the Royal Victoria and Albert Docks, was held on Wednesday evening, May 10th, at the Hotel Cecil, the Right Hon. Joseph Chamberlain, M.P., presiding. The company present numbered between 300 and 400, and included His Excellency Baron Whettnall (Belgian Minister), the Most Hon. the Marquis of Lansdowne (Her Majesty's Secretary of State for War), Sir Donald Currie, G.C.M.G., M.P., the Most Honourable the Marquis of Lorne, K.T., G.C.M.G., M.P., the Lord Chamberlain, Lord Lister, M.B., P.R.S., LL.D., F.R.C.S., Lord Strathcona and Mount Royal, G.C.M.G., D.C.L. (High Commissioner for Canada), Lord Loch, G.C.B., G.C.M.G., D.C.L., Right Hon. Lord Justice Vaughan Williams, Hon. Mr. Justice Grantham, Lord Basil Blackwood, Sir Henry C. Burdett, K.C.B., Viscount Duncannon, C.B., Sir R. G. W. Herbert, G.C.B., D.C.L., LL.D., the Hon. Sir Philip Oakley Fysh, K.C.M.G. (Agent-General for Tasmania), Lieut.-General Sir G. S. White, G.C.B., G.C.S.I., G.C.I.E., V.C., Admiral Sir Anthony Hiley Hoskins, G.C.B., Keishiro Matsui, (Japanese Legation), Rev. Brooke-Lambert, M.A., B.C.L., Sir Walter Peace, K.C.M.G. (Agent-General for Natal), Surg.-General Cleghorn, C.S.I., M.D., Alderman Sir Marcus Samuel, Dr. Stephen Mackenzie, Mr. Perceval A. Nairne (Chairman of Committee of Management), Mr. H. M. Stanley, D.C.L., LL.D., M.P., Hon. E. H. Whittenoom (Agent-General for Western Australia), Sir Wm. Priestley, LL.D., M.D., F.R.C.P., M.P., Hon. Dr. J. A. Cockburn (Agent-General for South Australia), Sir Gerald S. Vesey Fitz-Gerald, K.C.I.E., C.S.I., Mr. C. P. Lucas, Hon. J. C. Burns, Lord Ampthill, Mr. Sidney Buxton, M.P., Sir Alexander Grierson, Bart., Sir G. D. Taubman-Goldie, K.C.M.G., Sir. Wm. Corry, Bart., Colonel and Sheriff Probyn, Mr. Alderman and Sheriff Alliston, Sir C. Gage-Brown, K.C.M.G., Sir George Arthur, Bart., Mr. W. Garland Soper (secretary), Admiral E. S. Adeane, C.M.G., Major the Hon. W. Rowley, Sir Frederick Young, K.C.M.G., Sir M. M. Bhownaggree, K.C.I.E., M.P., Sir Frederick Bridge, Mus. Doc., Sir. W. R. Kynsey, C.M.G., Sir Wm. Des Vœux, G.C.M.G., Sir Wm. Robinson, G.C.M.G., Sir Clement Hill, K.C.M.G., C.B., Sir J. Wolfe Barry, K.C.B., Sir Leslie Falkiner, Bart., Sir Cavendish Boyle, K.C.M.G., Sir Jas. Lyle Mackay, K.C.I.E., Mr. B. L. Cohen, M.P., and Sir Somers Vine, C.M.G.

The usual loyal toasts having been honoured,

The Marquis of Lorne gave the toast of "The Navy, Army, and Auxiliary Forces."

Admiral Sir Anthony H. Hoskins replied for the Navy, and the Marquis of Lansdowne replied for the Army.

Mr. W. Garland Soper proposed "The Houses of Parliament," and Lord Loch replied on behalf of the House of Lords.

The Chairman, on rising to propose the toast of "The Establishment of the London School of Tropical Medicine, and the Enlargement of the Branch Hospital of the Society, in the Royal Victoria and Albert Docks," was very enthusiastically received.

Mr. Chamberlain's Speech.

Imperial Policy.

He said: I have now to propose to you the toast of the evening, "The London School of Tropical Medicine." I hope you will not be alarmed, and that you will not fear that I am about to lead you into the thorny paths of political controversy—(hear, hear)—when I say that we are met here to-night in order to promote Imperial policy. (Laughter and hear, hear.) I am sure that when our object is fully explained we shall have the hearty sympathy and support of men of every party and of every shade of politics. In saying that I do not rest myself merely on the statement that we have had on high authority recently that we are all Imperialist now. (Laughter and cheers.) I feel that I should not be right in dwelling too much upon that admission, especially in the case of recent converts —(laughter)—and that I am not entitled to expect that those who are, as it were, only catechumens in the Imperial Church should already be deeply rooted in every point of faith and doctrine.

England's Great Obligations.

But I think I am justified in saying that, whatever divergencies of opinion may exist upon such questions as the extension of the empire, the acquisition of new markets, and our relations with subject races, we are all agreed in desiring that the territories which are already under our control should be wisely, honestly, and justly administered, principally in the interests of the immense populations that have come under our influence. (Cheers.) In the discharge of this great obligation, the heaviest, perhaps, that in the history of the world has ever been thrown upon a single nation, we have to rely upon the courage, the energy, and, above all, the character of those of our fellow-countrymen whom we lend—and whom, unfortunately, we are often called upon to give—to the service of our dependencies. (Cheers.) Now, the British Empire contains, I suppose, roughly, something like three hundred millions of coloured people living mostly in tropical climates, and it is the government and control of the tropics which forms the principal part of the white man's burden, the immensity of which has caused some of our Statesmen to regard the future with anxiety akin to fear. I observe that Mr. Rhodes, in a recent speech, referred to a conversation that he had had with Mr. Gladstone, in which that great Minister had said that he was afraid lest the number of men qualified for Colonial administration should be insufficient for the work which an extended Empire involved. For myself, I agree with the reply which was made by Mr. Rhodes.

The British Character.

I believe that so long as the British character remains unchanged the opportunity of distinction, the prospect of adventure, and the love of responsibility which is deep-seated in our character will always prove sufficient temptation to secure that the supply shall be equal to the demand. (Cheers.) But, my lords and gentlemen, that does not relieve us from the obligation to see that every precaution is taken, and every provision made to prevent this splendid material, these potent instruments of our greatness, these props of our Empire, from being wasted and thrown away owing to the neglect of precautions which, if they were properly fulfilled, might save their lives or extend their usefulness.

The Insidious Attacks of Deadly Disease.

Now, in this great work of civilisation and government, our greatest enemy is not the hostility of savage chiefs nor the influence of barbarous customs, nor even the physical difficulties of countries in which primeval nature still holds full sway, but it is rather the insidious attacks of a deadly disease which weakens where it does not kill, and which shortens the lives and careers of many of the ablest and most energetic of those who go out to represent us in these countries. (Cheers.) It is impossible for anyone who occupies the position which I have the honour to fill not to be painfully impressed with the tragedy which is always being enacted; with the mortality which cuts off young men of great promise in the very outset of their career; which strikes down in their prime experienced administrators and able and conscientious officials; which even, when it is not fatal to individuals, at all events weakens the service by constant illness, disorganises the administration, and prevents anything like continuity of policy owing to the frequent breaks which are necessary in order that impaired health and strength may be restored. And the problem before us is manifold.

Finding a Cure.

We have to seek out and make clear the history and origin of these diseases. We have, if possible, to find a cure for them, and with those objects we have to extend the study of these tropical diseases, and to create, if it may be, a school of trained practitioners and investigators, so that in future scientific research may go hand in hand with practical medicine. (Cheers.) Meanwhile, we must not forget the necessity for meeting, as far as may be, the conditions of life in these tropical countries, for impressing upon all who go there the necessity for the observance of ordinary sanitary laws, and for securing for our sick countrymen the best possible attendance and the most skilful treatment. Now we have made some progress.

The Colonial Nursing Association.

Lord Loch has referred to the Colonial Nursing Association. We owe it to the initiative of one lady, Mrs. Pigott, herself the wife of a most able Colonial official, and to the committee which she has formed, that this association is substituting everywhere in our colonies trained and educated nurses for the native attendants, often careless, frequently more or less ignorant, to whom hitherto, and for too long, sufferers from these diseases have been confined. In the United Kingdom we have for a long time dispensed with the services of Mrs. Gamp and Betsy Prigg, and I think you will agree that their native representatives are still less desirable attendants in the sick room than their British originals. (Laughter.)

Lord Lister's Assistance.

Then, thanks very much to the sympathy and assistance of Lord Lister, to whom I desire to express the gratitude of all interested in this subject, and his colleague, Professor Foster, we have enlisted the aid of the Royal Society—of which, by the way, I am myself a most useless and unworthy member—to conduct an investigation into the origin and cause, and, if possible, to find a cure for, the malarial and blackwater fevers, which are, perhaps, the most deadly of all tropical diseases, and with the assistance and advice of the Royal Society, two able and capable investigators appointed by them and one by the Colonial Office are now engaged in an inquiry into the origin and transmission of those diseases; and after having made themselves acquainted with previous researches in Italy, and with the results of the inquiries and experiments of Dr. Ross in India, they have proceeded to Blantyre, where they will make a lengthened stay, and from there they will complete their work in West Africa, where these diseases appear in their most virulent forms.

A School Necessary.

But these investigations and inquiries, important as they are, would be absolutely incomplete and insufficient without such a school of tropical medicine as that which we are met to-night to promote. At this point let me express my gratitude to the medical adviser of the Colonial Office, Dr. Patrick Manson, and his predecessors, Sir Charles Gage Brown, Dr. Rowell, and many other distinguished medical men, owing to whose advice and suggestion in the first place, and practical labour afterwards, this institution has become possible, and, in fact, may already be said to be a success. I do not think it is necessary for me to dwell at any length on the necessity for such a school. I have been very glad to learn, in answer to a circular issued some time ago from the Colonial Office to the principal schools of medicine in the United Kingdom, that many of these great institutions will in future give special attention to tropical diseases, and we may hope, therefore, that a much larger proportion than formerly of medical men educated in this country will, at all events, have a thorough knowledge of the theory, origin, and treatment of these diseases. But, gentlemen, that is hardly enough. Something more is wanted in the shape of practical experience and clinical instruction, and that can hardly be satisfactorily afforded except in cases, as Lord Lister said the other day at Liverpool, in a tone which struck me as almost of enthusiasm, where there is a special supply of diseases.

The Albert Dock Hospital.

I do not know whether I ought to say that I am glad or sorry, but a full supply of those diseases is not likely to be absent from the hospital at the Victoria and Albert Dock. Under the scheme which has been proposed, the accommodation at that hospital is to be increased to fifty beds, and we have reason to believe those beds will almost always be accordingly filled with acute cases of tropical disease. When this enlargement has taken place, when the necessary school buildings have been erected, and also accommodation for the students expected to attend, the course of procedure will be something as follows:—

Selection by the Colonial Office.

The Colonial Office, and I believe the other great offices which have dependencies in the tropics under their charge, will give a preference in their selection of medical officers for those colonies and dependencies to the gentlemen who have gone through a course of special study in connection with the subject of tropical diseases in one of the great medical institutions of the country. This course of study may extend over two or three years, and may be given in any of the medical institutions of the United Kingdom. After that study has been completed, and the necessary certificates and degrees have been conferred, it will be expected that all the candidates whom we shall have selected shall take advantage of tropical schools of medicine, and practically learn the treatment of disease where it is possible to do so. In the first instance the London School will supply that requirement. It may be hereafter, I think probably at no distant date, in Liverpool and elsewhere we may possibly find centres where there is an equal supply of material for demonstration and instruction, and in that case it may not be necessary to require that this instruction shall be gone through, and the student may be able to complete his service previous to appointment in the institution in which he has received his education. Now this is not all that we propose to do or that the authorities of the hospital propose to do. If sufficient funds are provided they will undertake to provide a central museum and library, which will be of great importance in future investigation and research.

Travelling Scholarships.

They will do something more, something to which I personally attach the greatest importance; they will establish travelling scholarships, with which those best qualified to undertake such investigations will pursue researches into these various tropical diseases. This scheme has been widely welcomed and cordially approved. The India Office, the Foreign Office, the Colonial Office have all welcomed it with enthusiasm. The Imperial Government has made a large contribution towards the necessary expenses—(hear, hear)—the Colonies chiefly concerned have been appealed to and have liberally responded, and several of them have voted even larger sums than those which we asked. The King of the Belgians, that enlightened head of a great tropical State, has shown his interest in the undertaking by a handsome subscription, and has promised that he will take advantage of the facilities which the school will give.

The Co-operation of the Colonies.

Now, therefore, it remains for me to appeal to all who are interested, either directly or indirectly, in our Colonies to co-operate in a movement which will be fruitful of great results, whether to the Government of this country, or to the missionary enterprise which is carried on on behalf of other populations, or in the development of our trade and commerce. I must not, however, pass away from this without some reference to an interesting confirmation of our proceedings, showing, I think, that we are on the right path. It is said that imitation is the sincerest form of flattery, and I rejoice to say that no sooner was our scheme matured and published than the citizens of Liverpool, with the energy and liberality which always characterises them, hastened to create a school of a similar character in their city. We welcome their co-operation, and that of all others who may take a similar course. There can be no question of jealousy as between several institutions engaged in the same work. We hope that they will always keep in close touch with us. We shall share with them our respective investigations and discoveries, and I am sure that the more numerous the labourers in this field the sooner we shall reap the harvest and the more plenteous the harvest will be.

A Bountiful Harvest.

I think I am not too sanguine when I say that we have a right to expect a bountiful and beneficent harvest. Why should we despair? Is it too much to ask of science that it shall confer this great benefit upon mankind? When I look at what has been done by the efforts of science with respect to the plagues of the Middle Ages which devastated vast districts, and which had now ceased out of the land; at the fact that within our own time cholera and typhus have lost most of their terrors; when I remember what we have been told by

Sir A. Hoskins, and how in the West Indies the frequency and virulence of the epidemics of yellow fever have greatly diminished; when I know that colonies like Hong Kong, cities like Calcutta—which during the present century earned the evil reputation of being the white man's grave—have now become comparatively safe, I say there is no reason to despair that science may yet do something to lessen the unhealthiness of other settlements, and especially of those in Africa, thus remedying the greatest hindrance to the development of that vast continent.

### The Tropics Livable for White Men.

The man who shall successfully grapple with this foe of humanity and find the cure for malaria, for the fevers desolating our colonies and dependencies in many tropical countries, and shall make the tropics livable for white men, who shall reduce the risk of disease to something like an ordinary average, will do more for the world, more for the British Empire, than the man who adds a new province to the wide dominion of the Queen. All those who co-operate in securing this result, whether by their personal service or by some pecuniary sacrifice, will be entitled to share the honour and to add their names to the golden record of the benefactors of mankind. I propose to you "The London School of Tropical Medicine," coupled with the name of Sir Donald Currie, who has done so much to secure its complete success. (Cheers.)

The Secretary then read the list of donations. These included the following: The Colonial Office, £3,550; the India Office, £1,000; the Bishop of London (the Marriott bequest), £2,000; Admiral Coote, £500; the King of the Belgians, £200; Sir Thomas Sutherland, £500; Sir Donald Currie, £3,500. The London and India Docks Joint Committee had, he said, given the site upon which the new hospital and school would be built. (Applause.) Lord Strathcona and Sir Donald Currie would each give £500 to name a bed in the new hospital. The total amount received prior to the dinner was £14,700. (Loud applause.)

The Chairman said he was pleased to be able to announce the promise of Sir Henry Burdett to secure £300 a year for three years for a travelling scholarship.

Sir Donald Currie (vice-president of the society), in acknowledging the toast, expressed the warm gratitude of the committee of the society to the chairman for the eloquent manner in which he had advocated the cause of the hospital and of the new arrangements they were making in connection with the study of tropical disease.

Lord Lister gave "The Empire."

Lord Strathcona and Mount Royal made a suitable acknowledgment, after which "The Health of the Chairman" was proposed by Sir Donald Currie in a few well-chosen words. Mr. Chamberlain made a brief response, again expressing the pleasure with which he had taken up the cause that all present had at heart, and thanking them for the support which they had accorded to it.

---

### NORTH LONDON HOSPITAL FOR CONSUMPTION.

The annual festival dinner in aid of the funds of the North London Hospital for Consumption and Diseases of the Chest was held on May 10th, at the Hotel Cecil, Strand, W.C. Sir Henry Harben presided, and among those present were Mr. E. Bond, M.P., L.C.C., Mr. E. Brodie Hoare, M.P., Mr. Alfred Hoare, L.C.C. (hon. treasurer), Mr. J. S. Fletcher, L.C.C., Dr. F. P. Weaver, J.P., Dr. O'Connor, Dr. Macgregor, Mr. S. Figgis, Dr. Bunch, Dr. Johnston, Mr. B. A. Lyon (chairman of committee), Mr. G. C. Trewby, Colonel John Davis, A.D.C., the Rev. Evelyn Beaumont Burnaby, the Rev. Dr. P. H. O'Brien, Mr. H. J. Allcroft, J.P., Mr. Henry Stedall, Sr. Don Eduardo Toda, Con.-Gen. for Spain; Ilmo Sr. Don Nicasio Jaraulde, Spanish Fin. Del.; Dn. Roman Lopez, Dn. Francesco Segreda, Mr. W. J. Morton (secretary), and many others.

The toasts of "The Queen," "The Prince and Princess of Wales and the other members of the Royal Family," and "The Houses of Parliament," having been enthusiastically received,

The Chairman submitted the toast of the evening, "Prosperity to the North London Hospital for Consumption." He said they were present to discuss a new departure in connection with the hospital, namely, the open-air treatment of consumption. (Applause.) Some years ago the late Lord Playfair predicted that within a comparatively short period consumption would be as much a thing of the past as typhus fever. In the year 1870, with a population of 22,000,000, there were 3,297 deaths by typhus. In 1897, with 31,000,000, there were only 49. (Applause.) Therefore, typhus was practically stamped out. Lord Playfair had stated that the idea of hereditary consumption was a myth. In 1870 the deaths from consumption were 54,232, and in 1897 41,642. (Hear, hear.) The percentage of deaths from this disease to the population in 1870 was 2·41 per cent., and in 1897 1·34 per cent. (Applause.) That was what medical science had done, and he believed that if they supported the staff of this hospital in their new departure that Lord Playfair's prediction would come true within the lifetime of many of those present. He cited a case where a young lady, whose brother died of consumption in Africa, had been completely cured of this fell disease by the open air remedy. To carry out the new system increased expenditure would be necessary, and he hoped they would come forward liberally and help those connected with the hospital to stamp out a terrible disease which had been the curse of English life. (Applause.)

The toast was drunk with enthusiasm.

Mr. Alfred Hoare, L.C.C. (hon. treasurer), in reply, said that perhaps to Mr. Lyon (chairman of the committee) more than anybody was due the fact that the hospital had paid off its debt, and had risen to the stage of being the leading hospital for the treatment of consumption in this country, if not in the world. (Applause.) As treasurer he hoped they would entrust him with a very heavy burden. (Laughter and applause.)

Mr. B. A. Lyon (chairman of the committee) also responded. He said that some years ago they had only 39 beds in the hospital, together with a very precarious income of between £2,000 and £3,000 a year, while at the present time they had 80 beds all full and a sufficient income to pay their way. They had every reason to speak hopefully regarding the ultimate success of the open-air treatment. (Applause.)

Mr. Edward Bond, M.P., proposed "The Medical Staff," and in doing so remarked that the lengthened services of many of the distinguished men who formed the medical staff was a testimony to their distinction and to the excellency of the hospital itself. Dr. O'Connor had served the institution for no less than 16 years. In his opinion simplicity was a great thing, and it was embodied in the open-air treatment. (Applause.)

The toast was cordially received.

Dr. O'Connor, in acknowledging, said that while typhus fever for all practical purposes had been stamped out, they had to grant with regret that, so far as the stamping out of consumption was concerned, they had not been entirely successful. The open-air remedy was no means a new departure. Some of the members of the hospital staff had been advocating that method of treatment for some considerable time, but it was not until recently that they had had an opportunity of carrying it out in detail to anything like approaching their own satisfaction. The open-air treatment involved increased expenditure in connection with the nursing staff and maintenance; and, therefore, they had a direct reason for enlarging their subscriptions on the present occasion. Medical men in Spain for ages past had been of the belief that consumption was a contagious disorder, and it was a disorder which was best combated by exposure to the pure open air and as much sunlight as could be obtained. The capacity of the chest had been considerably increased during the last six or seven years, and he put it down to the almost universal practice of bicycle riding, which he strongly recommended. In dealing with poor afflicted sufferers from consumption great advantage was to be derived from an institution such as they possessed at Hampstead. (Applause.)

Mr. W. J. Morton (secretary) read a list of subscriptions to the amount of £5,312, which included £2,786 collected by the chairman and £1,568 collected by Mr. B. A. Lyon.

The Chairman then announced that he had received a cheque from a gentleman, who preferred to remain anonymous, for an additional £4,000. (Loud applause.) The total contributions were, therefore, £9,312. (Renewed applause.)

With the toast of "The Chairman," proposed by Mr Samuel Figgis, and suitably acknowledged by Sir Henry Harben, the proceedings terminated.

### HOSPITAL FOR DISEASES OF THE SKIN.

The annual meeting of the governors and subscribers of the Hospital for Diseases of the Skin was held at the institution on Wednesday, 10th inst., Mr. Waren Tay, F.R.C.S., presiding. The report of the committee of management showed that during the past year 3,392 new patients had been admitted, as compared with 4,321 in 1897; the total attendances amounted to 15,653, an increase of 1,174; 26 were treated as in-patients, whose average stay in the hospital was 34 days. The total number of patients treated since 1841 was thus raised to 361,787. The committee drew special attention to the large increase in the work of the out-patient department. The certified accounts for the year showed that the ordinary income amounted to £929, and the ordinary expenditure to £1,022. There had been no extraordinary expenditure. The committee add that the diminution of annual subscriptions under which the hospital is now labouring is most serious, and is causing them deep anxiety, as the strain upon its financial resources has been very great. They appeal for increased support to prevent the possibility of the work being curtailed, feeling assured that ere long the institution will be placed on a self-sustaining basis. The report was adopted. The resignation of Dr. Payne was received with regret, and he was elected consulting physician. Three new trustees were appointed, viz., Sir James Dick, Colonel C. K. T. Marshall, and Mr. Louis Sebastian, and the committee for the new year having also been elected, the proceedings closed with the customary votes of thanks to the staff, and to the chairman for presiding.

### CHELSEA HOSPITAL FOR WOMEN.

The annual meeting of the Governors of the Chelsea Hospital for Women was held on Thursday, 11th inst., at the institution, Fulham Road, Mr. Robert J. Beadon presiding in the absence of Lord Glenesk, who was to have taken the chair, and who subsequently arrived. The twenty-eighth annual report of the council, which was read by Mr. Herbert J. Jennings, the secretary, showed that the work of the hospital during 1898 had been of a most satisfactory character. It stated that a comparison of the medical statistics for the year with those for 1897 showed an advance in the usefulness of the institution, and a comparison of the annual subscriptions (£1,642 against £1,607) made it evident that the hospital had been steadily progressing. The grants made by the Sunday Fund to the hospital and its convalescent home (which is situated at West Hill, St. Leonards) for the past year amounted to £311, as against £306 for 1897; whilst the amount received by the two institutions from the Saturday Fund was £151, as against £140 for the previous year. The council record how much they value the generous appreciation of the committee of the Prince of Wales's Fund of the needs of the hospital and its convalescent home, and express their gratitude for grants amounting for 1898 to £350, as against £306 for 1897. The council had at last secured the much-needed accommodation for the nursing staff in No. 13, Neville Street, almost opposite the hospital, a fifty-six years' lease of which had been purchased for £1,600. Each nurse had been provided with a separate bedroom. The completion of this purchase and adaptation involved an outlay of nearly £2,000, and only £1,100 of that sum having been in hand, a considerable deficiency was left to b made up. The council, in compliance with the urgent representation of the medical staff, were arranging to carry out further improvements, the cost of which would be £3,000. The accounts showed that, excluding a balance of £1,264 from 1897, the total ordinary income for the year was £4,567, and the extraordinary income £1,661, making together £7,492. The total ordinary expenditure was £4,889, and the total extraordinary expenditure £725, giving a credit balance of £1,880. The number of patients admitted during the year was 671; new out-patients totalled 2,884; and the attendances of all patients, 10,479. The work of the Convalescent Home was separately treated, the total of patients admitted having been 249, against 231 in 1897. Its annual subscriptions had substantially increased, and its ordinary income was £661, and its extraordinary income a legacy of £450; the ordinary expenditure was £551, and the extraordinary £9 only, giving a credit balance of £603. The council, with Lord Glenesk as chairman, was elected on the motion of Dr. Fenton, seconded by Mr. J. E. Kitchingman.

At this stage of the meeting Lord Glenesk arrived, and briefly alluded to the satisfactory nature of the report, and touched upon its main features. Votes of thanks to the honorary medical staff, other honorary officers, and to Lord Glenesk and Mr. Beadon for presiding, concluded the proceedings.

### BELGRAVE HOSPITAL FOR CHILDREN.

### Transfer of the Institution to Kennington.

In view of the need in South London for further hospital accommodation for children, the governors of the Belgrave Hospital (founded in Pimlico in 1867) have decided to transfer that institution to a site which has been secured at Kennington, and on Friday, 12th inst., a public meeting was held at the Mansion House in support of the scheme and to consider resolutions for its approval.

The Duke of Westminster presided in the absence of the Lord Mayor, and in opening the proceedings said he had been connected with the Belgrave Hospital for many years. It consisted of two very inadequate houses, which, however, had served as a hospital for 30 years, and had done admirable work. The committee for a long time had been considering whether it would not be better to remove the institution altogether and build one on another site which would be entirely adequate for its objects. He had offered the committee a site, but it had not proved a suitable one, and so they now proposed to remove it to South London, a district which it was found was very inadequately supplied with hospitals, especially children's hospitals. In fact, the difference between the north and the south of the Thames in this respect was enormous; the existing accommodation in hospitals for children was represented by one cot for 12,560 head of population on the south side, as compared with one cot for 3,523 on the north side. So that they had a very good claim for the support of their proposal to remove the hospital to the south side of the river. The committee had therefore decided to remove the Belgrave Hospital, and had secured an admirable site in the Clapham Road, close to Kennington Park. They had something like £5,000 in hand, but they required altogether £50,000, a portion of which would, however, go towards endowing the institution. He believed there was plenty of money about for such objects if it could only be obtained at the right time, and instanced the remarkable response made to the appeal for the building of the Gordon College at Khartoum. He knew of nothing more deserving aid than the cause of the hospital they were discussing. He moved a resolution to the effect that there was urgent need for the increase of hospital accommodation for children in South London.

The Bishop of Rochester, in seconding the motion, said he was present to set forth the greatness of the need of the proposed change rather than to represent the origin of the movement. South London was the most needy district in his diocese, and it was marvellous how little was known of the conditions prevailing in the group of cities on the south side of the Thames. That morning he had seen on a newspaper placard, "A town built since Monday last," and in the growth of their population they came much nearer to that in South London than that meeting would suppose, for South London—one-third of the whole of the metropolis—was increasing its population at the rate of 26,000 a year, compared with 14,000 for North London. It was a case of "out of sight, out of mind." They had no City, no West-end, and no centre of activity, and there was not, as in North London, the same power of bringing wealth to bear and of giving it an initiative. As to their hospital accommodation for children, they in the South had only two, whilst in North London there were eleven. If they took the need for the increased provision in South London they would find the case a strong one, for it contained many districts where the conditions were prejudicial to life and health, and especially conducive to infant mortality, for from some figures one of his clergy had supplied him with it would appear that, whilst in Stoke Newington, in North London, only 108 babies per 1,000 die, yet in Newington, a South London district, the infant mortality was 173 per 1,000. They could not fall back upon the old theory that only the parent was responsible for the life of the child. All must share, and they ought to feel bound and willing to do all they could to further the transference of the Belgrave Hospital.

The Rev. Bernard Snell, in supporting the resolution, extended a welcome to the scheme, and said he considered it was not that the public were not willing to help such a cause, but that they had no knowledge of the facts. Child life had hard times down their way, and they would congratulate themselves that their new hospital was not a new and untried institution. It was an ideal proposal, and, therefore, doubly welcome.

The resolution was unanimously carried.

Dr. Farquharson, M.P., moved a second resolution to the effect that the meeting approved generally of the scheme of transfer.

This was seconded by the Hon. Evelyn Hubbard, M.P., who, in doing so, expressed the hope that the Government, whilst it was dealing with the London Government Bill, might see its way to accede to the gentle pressure which was going to be put on Mr. Balfour to do something for the London hospitals by exempting them from rating. He was authorised to say that they could build the central block of the hospital, including administration accommodation and two floors of wards, for £10,000, and an out-patients' department for another £5,000, so that with that amount they could make a good start.

Mr. Clinton T. Dent supported the motion, and it was adopted.

The proceedings terminated with votes of thanks to the Lord Mayor for the use of the Mansion House and to his Grace for presiding.

